# Emerging roles of extracellular vesicles in neurodegenerative disorders: focus on HIV-associated neurological complications

**DOI:** 10.1038/cddis.2016.336

**Published:** 2016-11-24

**Authors:** Guoku Hu, Lu Yang, Yu Cai, Fang Niu, Frank Mezzacappa, Shannon Callen, Howard S Fox, Shilpa Buch

**Affiliations:** 1 Department of Pharmacology and Experimental Neuroscience, University of Nebraska Medical Center, Omaha, NE, USA; 2 School of Medicine, University of Electronic Science and Technology of China, Chengdu 610054, China

## Abstract

Exosomes are membrane-enriched extracellular vesicles with a proposed diameter in the range of 30–100 nm. They are released during both normal homeostasis as well as under pathological conditions by most cell types. In recent years, there has been robust interest in the study of these vesicles as conduits for the delivery of information between cells in both analogous as well as disparate tissues. Their ability to transport specialized cargo including signaling mediators, proteins, messenger RNA and miRNAs characterizes these vesicles as primary facilitators of cell-to-cell communication and regulation. Exosomes have also been demonstrated to have important roles in the field of cancer biology and metastasis. More recently, their role in several neurodegenerative disorders has been gaining increased momentum as these particles have been shown to promote the spread of toxic factors such as amyloid beta and prions, adding further validity to their role as important regulators of disease pathogenesis. This review briefly summarizes current findings and thoughts on exosome biology in the context of neurodegenerative disorders and the manipulation of these particles for the development of potential therapeutic strategies.

## Facts

- Exosomes are globular, membrane-bound extracellular nanovesicles (30–100 nm in diameter) that are released by almost all types of cells.
- ARF6 and PLD2 have important roles in extracellular vesicle release through the regulation of the budding of ILVs into MVBs.
- Extracellular vesicular molecules (including ADAM17, TNF*α* and Nef) released from HIV-infected cells induce activation, apoptosis and HIV susceptibility in the recipient cells.
- Extracellular vesicles released from CD8^+^ T cells contain antiviral membrane-bound factors that inhibit HIV-1 transcription.

## Open questions

- Are HIV proteins such as Tat /gp120 released in the extracellular vesicles and if so, do they disseminate CNS toxicity?
- What is the role of EVs in propagation of pathogenic proteins in the neurodegenerative disorders?
- How can extracellular vesicle therapeutics be applied in the context of neurodegenerative diseases?

Cellular cross talk underlies most pathological conditions including those within the central nervous system (CNS). Although various factors have been identified as instigators of disease pathogenesis, it is now becoming clear that unrestrained neuroinflammation and, subsequent cellular toxicity are the key hallmark features of various neurological disorders. In this light, the notion that disease pathogenesis may be accelerated or mediated by exosomes and their associated cargos is recently gaining momentum. Exosomes are globular; membrane-bound extracellular nanovesicles (30–100 nm in diameter) that are released by almost all types of cells during normal cellular functioning and specifically, in response to cellular stressors. These small vesicles originally thought to contain 'junk' cellular debris were first described by Trams *et al.*^1^ when they observed smaller membrane-bound vesicles within the larger endosomes (later termed multi-vesicular bodies (MVBs). An electron micrographic study related the exosomes to sheep reticulocytes.^2^ The release of these small vesicles into the extracellular environment was proposed as a mechanism by which reticulocytes could secrete transferrin receptor. This proposed mechanism was further supported by *in vitro* analysis of sheep reticulocytes, which demonstrated the selective loss of certain proteins from maturing cells.^3^ An understanding of the role of exosomes in various cell types has evolved greatly. They are no longer viewed as waste bags; instead, exosomes are thought to have an important role as cargo-carrying vesicles mediating communication among different cells and tissues including the CNS.^4^ Exosomes are known to carry nucleic acids (RNA, microRNAs (miRNA) and DNA), functional proteins (including those of viral origin) and other cellular products. In the literature extracellular vesicle (EV) subtypes have often been given names such as exosomes, microvesicles, ectosomes or microparticles based on their biogenesis, physical characteristics (such as size), or function.

A growing body of evidence suggests the involvement of exosomes in many neuroinflammatory diseases. These small vesicles are important in CNS communication as most CNS cells secrete these particles.^4^ Cell–cell communication via exosomes can be envisioned to have an important role in pathogenesis through their ability to transmit disease-causing agents from one cell to the other. Indeed, exosomes have been associated with numerous neuroinflammatory diseases including Parkinson's, Alzheimer's and Creutzfeldt–Jakob diseases. Further research into the role of these vesicles in disease progression is important for the development of effective preventative and therapeutic options. The focus of this review is to examine the role of exosomes in the progression of various neurodegenerative disorders.

## Exosome Cargo

**Figure 1:**
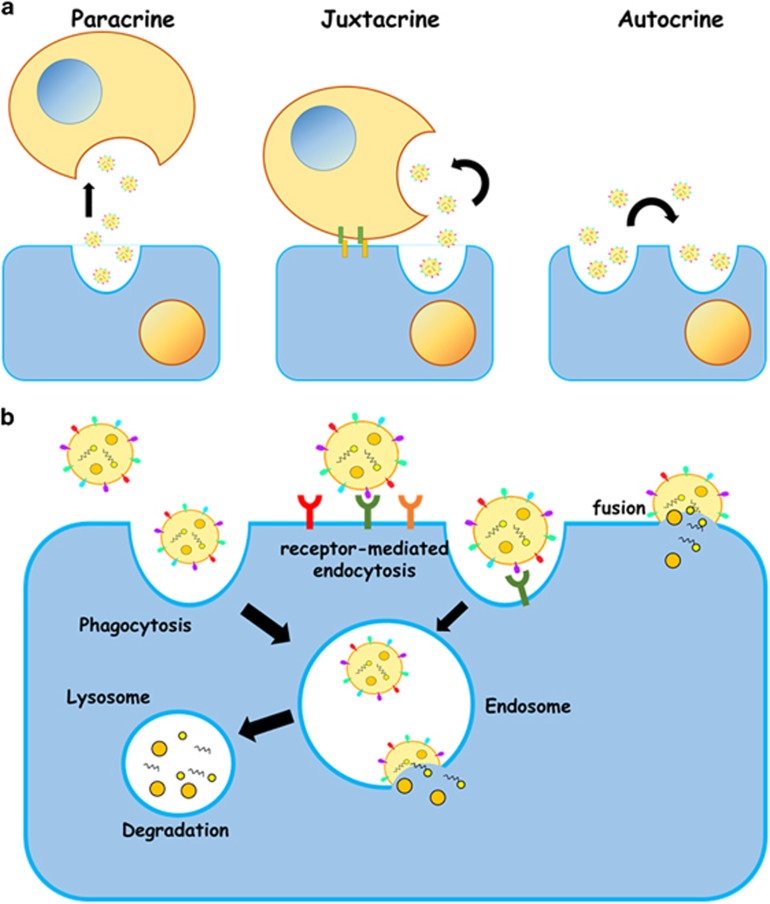
(**a**) Exosomes delivery via various signaling pathways. Exosomes can deliver molecules to distant cells (paracrine), adjacent cells (juxtacrine) or neighboring cells (autocrine). (**b**) The uptake of exosomes into a target cell occurs by one of the three mechanisms: phagocytosis, receptor-mediated endocytosis (RME) or direct fusion of exosomes with the plasma membrane of the recipient cell

Exosomes are generated via inward budding of the late endosomal membrane with the newly formed intraluminal vesicles (ILVs) destined for one of the two outcomes: either the late endosome merges with a lysosome, which degrades the ILVs along with their cytoplasmically derived cargo or the late endosome binds to the plasma membrane and releases the ILVs as exosomes with their cargo into the extracellular environment. Specifically, lysosome-directed vesicle formation occurs via the endosomal sorting complexes required for transport (ESCRT) machinery, whereas exosome budding is directed by the sphingolipid ceramide on the membrane of the endosome.^5^ Recent studies have shown that the small GTPase ADP ribosylation factor 6 (ARF6) and its effector phospholipase D2 (PLD2) regulate exosome release.^6^ ARF6 and PLD2 function together to regulate the budding of ILVs into MVBs.^6^ After formation of ILVs, exosomes are released from the cell upon fusion of the late endosome with the plasma membrane. These vesicles are then free to carry their cargo throughout the surrounding environment and are associated with juxtacrine, paracrine and endocrine uptake in the host tissue (see the detailed reviews discussing exosome biogenesis:^7, 8^ (Figure 1a). The specific composition of an exosome largely depends upon the originating cell and can vary widely depending on the cellular and environmental factors. Large-scale proteomic and phosphoproteomic studies of exosomes derived from various cell types suggest that these vesicles shuttle a wide array of biologically relevant molecules, including lipids, carbohydrates, RNAs and proteins.^9^ For example, the study from Knepper's group identified 1132 proteins contained within exosomes isolated from urine. In addition, unique phosphorylation sites have also been identified on exosomal proteins.^10^ Interestingly, miRNAs are abundantly present in the exosomes.^11^ MiRNAs regulate gene expression at the post-transcriptional level by binding to the 3'-UTR and/or the coding regions of their target mRNAs.^12^ In fact, hundreds of miRNAs have been found in exosomes.^13^ Many cell types, including reticulocytes, epithelial cells, neurons and tumor cells, have been reported to deliver exosomal miRNAs to recipient cells.^14, 15^ In addition, Epstein–Barr virus (EBV)-infected cells have been shown to secrete exosomes containing EBV-encoded miRNAs.^16^ Importantly, exosomal miRNAs can repress mRNAs in target cells and subsequently influence target cell function. Furthermore, these exosomal miRNAs have been implicated in a number of cellular processes and human diseases including cell migration, cell differentiation, cell viability, aging, neurodegeneration, cancer and immune disorders.^8^ These studies support the notion that exosomes obtained from body fluids have the potential to serve as biomarkers of disease development and/or progression. In this regard, Witwer *et al.*^17^ have suggested the need for standardization of specimen handling, appropriate normative controls, and isolation and analysis techniques for EVs/exosomes to facilitate comparison of results.

## Mechanism(s) of Exosome Interactions with Recipient Cells

A primary function of exosomes is their ability to deposit their cargo inside a recipient cell. Although many roles of exosomes have been extensively reported in the literature, detailed interactions between target cells and exosomes remain to be elucidated. Currently, it is hypothesized that the uptake of exosomes into a target cell occurs by one of the three mechanisms: phagocytosis, receptor-mediated endocytosis (RME) or direct fusion of exosomes with the plasma membrane of the recipient cell^15, 18, 19^ (Figure 1b). The latter mechanism involves the release of exosomal cargo directly into a cell following the fusion of the exosomes with the plasma membrane of the recipient cell. RME, on the other hand, consists of binding of exosomal surface proteins to proteins on the plasma membrane of recipient cells, thereby facilitating the targeting of exosomes to distinct cell types. The exosomes can then either fuse directly with the plasma membrane or follow a different endocytic pathway consisting of fusing with the delimiting membrane of an endocytic compartment (i.e., endosome, lysosome, etc.).^20, 21^

The uptake of exosomes by RME in any given cell is largely dependent on the exosomal surface protein and lipid composition, which in turn, is primarily based on the type and condition of the secreting cell. The types of exosomal surface proteins and the kinds of receptors on the target cell determine the interaction between the exosome and the recipient cell. In addition, it has been hypothesized that lipid receptors could also have a vital role in exosome recognition by the target cells. For example, phosphatidylserine (PS) on exosomal outer surface can interact with PS receptors, TIM1 and TIM4,^22^ inducing accumulation of neutral lipids in the recipient cells.^23^ In regard to the role of proteins in this uptake, an elegant study by Escrevente *et al.*^24^ has shown that pretreatment of exosomes and recipient cells with a broad specificity protease K significantly decreased exosomal uptake efficiency. This study concluded that proteins from both exosomes and cells were required for uptake.^24^ It has also been shown that heat-shock proteins such as Hsp90/Hsp70, which are chaperone proteins enriched in exosomes can interact with receptors such as low-density lipoprotein receptor-related protein 1 (LRP1), and that this interaction is critical for recognition of exosomes by target cells.^25^ These findings further validated the notion that the interaction between exosomal membrane proteins and recipient cell-surface proteins is fundamental for the uptake of exosomes via RME. Upon further inquiry, it was shown that blocking CD9 and CD81 tetraspanins, which are commonly expressed on exosomes, resulted in a significant decrease in the exosomal uptake efficiency by dendritic cells.^26^

It has been demonstrated that phagocytic cells including RAW 264.7 macrophages and U937 monocyte-derived macrophages (MDM) internalize exosomes via phagocytosis.^27^ More specifically, Feng *et al.*^27^ demonstrated that macrophages internalized exosomes more efficiently than other non-phagocytic cells. Using electron microscopy, it was observed that exosomes remained localized on the surface of non-phagocytic cells, whereas exosomes in the vicinity of phagocytic cells were enveloped into large cellular extensions. Furthermore, the colocalization of fluorescent phagocytic tracers and the PKH26-dyed exosomes further confirmed that phagocytosis was the primary exosomal uptake mechanism employed by the macrophages. Furthermore, actin polymerization, which is necessary for phagocytosis, when inhibited by either cytochalasin D or latrunculin B, resulted in significantly reduced efficiency of exosomal uptake. This further reinforced the idea that phagocytosis is an important mechanism for exosome internalization in the macrophages and likely also in other phagocytic cell types.

It has also been documented that oligodendroglia-derived exosomes can be taken up by microglia via macropinocytosis.^28^ Furthermore, the authors also demonstrated that oligodendroglia-derived exosomes colocalized with Lamp1, a lysosome or late endosome marker, indicating thereby that an endocytic pathway was involved in the uptake of these exosomes. In order to deduce that macropinocytosis was indeed the mechanism in question, the microglia were treated with several reagents that interfered with specific steps in the macropinocytosis pathway. Following inhibition of macropinocytosis, microglial uptake of exosomes was inhibited, thereby underscoring the role of macropinocytosis in internalization of exosomes by microglia.^28^

## HIV Budding and Exosome Biogenesis

**Figure 2:**
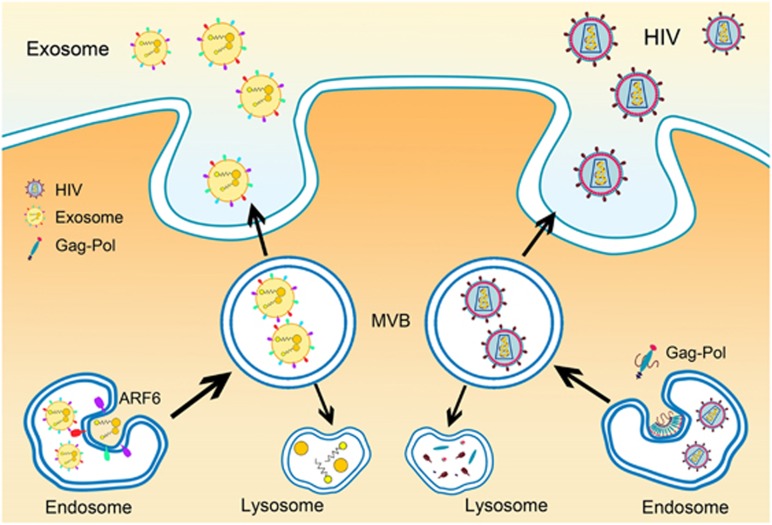
HIV budding is mediated by the exosome/microvesicle biogenesis pathway. HIV budding (left) and exosome release (right) share similar pathways. HIV Gag binds the viral RNA and drags it into the cytoplasmic face of intracellular vesicles, then the virus particles bud into the MVB. Subsequently, the virus-containing vesicles traffic to and fuse with the cell membrane, resulting in the release of virus particles

HIV acquires its envelope and propagates infection by budding through the limiting membranes of infected cells. HIV usurps a cellular pathway – that of formation of ILVs into MVB, to facilitate budding, indicating thereby that HIV budding and exosome release share a common mechanism. Indeed, published data on HIV-infected macrophages demonstrate the presence of HIV-containing vacuoles in macrophages that are reminiscent of MVBs.^29^ Using human MDMs, the study by Nguyen *et al.*^30^ revealed that the host protein profile of macrophage-derived exosomes and that of the HIV particles have a strong concordance, supporting the hypothesis that retroviral budding results from the exploitation of a pre-existing cellular pathway of intercellular vesicle trafficking. Proteomic analyses revealed that MDM-derived HIV virions contained 26 of 37 cellular proteins previously found in exosomes, consistent with the idea that HIV uses the late endosome/MVB pathway during virion budding from macrophages.^21^ In addition, Bieniasz *et al.*^31^ demonstrated that ESCRT components are recruited by HIV gag at the plasma membrane site of HIV budding. Nabhan *et al.*^32^ revealed a role for ESCRT components in ectosomes budding at the plasma membrane. It is thus speculated that the budding of virus and EVs could utilize similar cellular pathways at the plasma membrane and inside the MVBs. An excellent review on the similarities between EVs and viral entry has been published and could provide further insights on this phenomenon.^33^ Furthermore, it is also interesting to note that the HIV-1 virion has a similar size to that of the exosomes, an evidence supporting a similar origin of the two. This, however, also poses a potential confound for the isolation of exosomes from HIV-1-infected materials.^34^ In addition, studies on T cells and exosomes have been inconsistent and confounding. For example, some studies implicate that HIV-1 budding does not involve either endosomes or exosomes,^34, 35, 36^ whereas other reports have shown that HIV-1 budding from T cells is closely associated with exosomes.^37^ In this regard, Park and He^34^ have shown that high-speed centrifugation with 20% sucrose cushion during the last step can yield exosome-free HIV-1 virions compared with centrifugation only. Herein the authors provided a technical platform that could be employed to define the relationship between exosome biogenesis and budding of HIV-1.^34^ Though the small GTPase ARF6 and its effector PLD2 have an important role in exosome release, ARF6 is not involved in HIV-1 budding.^6^ Early studies on HIV budding demonstrated that loss of the viral envelope Gag p6 domain caused a severe defect in virus budding.^38^ However, recent studies showed that the N-terminal 433 amino acids of HIV Gag-Pol were sufficient to cause budding from cells, supporting the hypothesis that HIV budding was mediated by the exosome/microvesicle biogenesis pathway.^39^ In other cell types such as the dendritic cells, exosomes can be internalized and transfer signaling molecules to the recipient cells. HIV-1 particles exploit this exosome-dissemination pathway to spread infection to the dendritic cells, thereby underscoring a potentially new viral dissemination pathway (Figure 2 and Table 1). Taken together, it remains an exciting question of whether HIV-1 budding and exosomal biogenesis are closely related processes and/or whether they mutually influence the respective processes.

## HIV Infection Alters Exosomal Release and Composition

**Figure 3:**
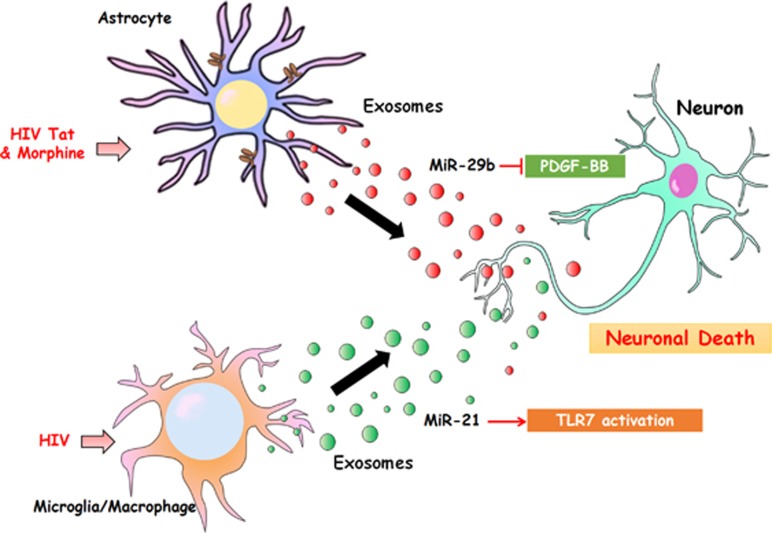
Exosome released from astrocytes treated with morphine and Tat carry miR-29b, which can be taken up by neurons, resulting in neuronal death. MiR-21 in EVs leads to neurotoxicity via the TLR7 signaling pathway

Both exosome release as well as exosomal composition are regulated through cell signaling pathways that are activated by many factors, including but not limited to, HIV infection and subsequent immune activation. For example, it has been shown by Kadiu *et al.* that exosome numbers are increased in MDMs following HIV-1 infection.^40^ Furthermore, HIV-1 was shown to accelerate infection and viral dissemination by surrounding itself with exosomes.^40^ HIV infection not only affects exosome release, but also impacts exosomal cargo. Indeed, large-scale proteomics studies have revealed that compared with uninfected cells, exosomes released from HIV-1-infected cells harbor distinct regulatory molecules and are composed of a unique and quantitatively different protein signature.^41, 42^ Fourteen proteins out of 770 were identified to be differentially expressed in the exosomal fractions of HIV-1-infected cells compared with the uninfected cells. Three immunomodulatory molecules included ADP-ribosyl cyclase 1 (CD38), l-lactate dehydrogenase B chain and Annexin A5.^41^ Recent studies have found that HIV-1 RNAs, such as HIV miRNA TAR, can also be incorporated into exosomes released from HIV-infected cells.^43, 44^ Furthermore, TAR RNA is sorted into exosomes in a chromosome region maintenance 1-dependent manner.^44^ Importantly, exosomal TAR inhibited apoptosis by downregulating Bim and Cdk9 protein levels in recipient cells,^44^ and stimulated proinflammatory cytokines, including IL-6 and TNF-*β* in primary macrophages.^45^ In a separate study, it was also shown that specific miRs such as miR-29b are transported via the EVs from HIV Tat and morphine-treated astrocytes to neurons and that, this transfer resulted in downregulation of PDGF-B (miR-29b target) in neurons, leading to neuronal apoptosis^46^ (Figure 3). A recent study by Yelamanchili *et al.*^47^ has also suggested increased expression of miR-21 in EVs derived from SIV-infected brains compared with the uninfected controls. Herein the authors demonstrated that in the brains of macaques with SIV-encephalitis, EV-miR-21 from donor macrophages/microglia resulted in neurotoxicity via activation of a TLR7-dependent downstream cell death pathway^47^ (Figure 3). HIV-1 infection of MDMs resulted in significant upregulation of a distinct class of miRNAs in exosomes isolated from the infected cells.^48^ Secretion of various HIV proteins has also been reported in the exosomes from infected cells. Specifically, the viral proteins Nef and Gag have also been found in released exosomes.^37, 49, 50, 51, 52, 53, 54^ It was also shown that Nef-containing exosomes were able to fuse with HIV-1 virions and deliver functional Nef to the virions as well as fuse with bystander cells to induce apoptosis in these cells.^50^ In addition, it has been shown that HIV Tat is also secreted and is present in exosomes derived from Tat-expressing astrocytes, and HIV-infected cells, and can be taken up by the neurons, leading to neuronal injury and death.^55^ A recent study demonstrated that exosomes are also enriched in cytokines in the plasma of HIV-positive individuals relative to the negative controls.^56^ These studies provide a basis for exosomes as biomarkers for HIV infection. Elegant work from the study by Sampey *et al.*^137^ using hydrogel nanotrap particles as affinity baits, has demonstrated the capture of HIV-1 virions, HIV proteins and exosomes-containing TAR-RNA in the patient serum. This could have ramifications for the future development of HIV diagnostics.^57, 58^ Further studies aimed at exploring the role of exosomes in the potentiation of HIV-associated complications are important for the development of effective therapies against these disorders.

## Functional Effects of EVs in the Context of HIV Infection

**Figure 4:**
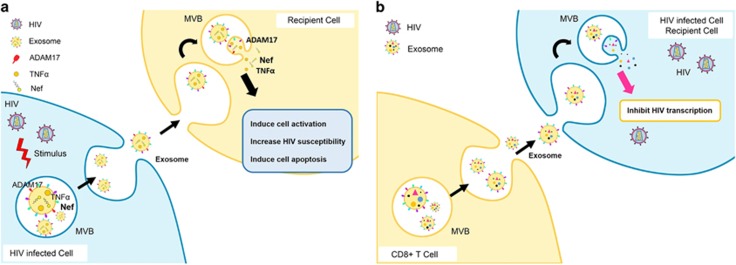
The exosome response to HIV infection. (**a**) Exosomes from HIV-infected cells contain various molecules including ADAM17, TNF*α*, Nef and so on. These molecules upon entering the recipient cells then induce the target cell activation, apoptosis and HIV susceptibility. (**b**) Exosomes released from CD8^+^ T cells contain an antiviral membrane-bound factor that inhibits HIV-1 transcription

It has been reported that the release of exosomes from HIV-1-infected lymphocytes is associated with HIV-1 replication in co-cultured quiescent CD4^+^ T lymphocytes.^59^ Exosomes from HIV-1-infected cells expressing a functionally defective viral mutant can still induce cell activation and lead to HIV-1 susceptibility in unstimulated CD4^+^ T lymphocytes.^60^ A Nef domain, 62EEEE65 acidic cluster, has been identified as a contributor of these effects.^59, 60^ Furthermore, active ADAM17 associates with exosomes from HIV-1-infected cells and induces HIV-1 replication in resting CD4+ T lymphocytes, thus stimulating viral spread.^59, 60^ (Figure 4a). In addition, exosomes released from HIV-1-infected cells can also impact many cellular processes in the recipient cells including proliferation and apoptosis.^41^ Specifically, exosomal Nef can enter the target cells and cause activation-induced cell death in resting CD4^+^ T lymphocytes.^51^ This is not surprising since Nef itself has been shown to induce dramatic dysregulation of cellular and exosomal miRNAs in human monocytic cells.^61^ Interestingly, exosomes purified from a transformed CD8^+^ T-cell line have been shown to an antiviral membrane-bound factor that inhibits HIV-1 transcription in both acute and chronic models of infection^62, 63^ (Figure 4b). Furthermore, a recent study demonstrating the association of cytokines with exosomes in the plasma of HIV-seropositive individuals suggests the role of exosomes in inflammation and viral propagation via bystander cell activation.^56^ In summary, alterations in exosomal cargo, following HIV infection contribute to HIV pathogenesis via multiple mechanisms including viral dissemination, cell apoptosis and inflammation.

## Alzheimer's Disease and EVs

Alzheimer disease (AD) is the most common neurodegenerative disorder with 46.8 million people affected worldwide, clinically characterized as an ongoing cognitive impairment.^64^ Aggregation of hyperphosphorylated tau in the neurofibrillary tangles and accumulation of amyloid beta (A*β*) plaques are the two salient pathological features of AD.^65^ Accumulating evidence suggests the involvement of EVs in the pathogenesis of AD.^66, 67, 68^ Many reports also implicate spread of the pathogenic AD proteins via the EV cargo.^67, 69, 70^ For example, exosomal proteins such as Alix and flotillins have been reported to be localized within the amyloid plaques in the brains of the Tg2576 mice (AD model) as well as in the postmortem tissues of human AD patients.^69, 70^ In support of this is also another clinical report indicating upregulation of AD pathogenic proteins including P-T181-tau, P-S396-tau and A*β*_1–42_ in the plasma exosomes isolated from AD patients, compared with cognitively normal-matched healthy individuals.^67^ Furthermore, mechanistic studies have also implicated that both cleavage and endocytic transportation of amyloid precursor protein (APP) have cardinal roles in packaging A*β* into exosomes for dispersion.^70, 71, 72^ A*β* is a cleaved product resulting from the cleavage of APP by the *β*- and *γ*-secretases.^73^ A finding by Rajendran *et al.*^70^ demonstrated that A*β* was sorted into MVBs in both HeLa and N2a cells following *β*-cleavage in the early endosomes. Interestingly, Sharples *et al.*^72^ also reported that inhibition of *γ*-secretase stimulated *α*- and *β*-cleavage, leading in turn, to C-terminal fragments of APP (APP-CTFs) in the exosomes. Furthermore, *γ*-secretase has also been shown to be required for the clearance of APP-CTFs from the endocytic recycling compartments, which constitute a series of perinuclear tubular and vesicular membranes, that regulate recycling of APP-CTFs to the plasma membrane.^74, 75^ In addition, deficits in retromer, a multimeric complex that mediates retrograde protein transportation from endosome to the trans-Golgi network, has also been shown to promote amyloidogenic APP processing by enhancing interactions between APP and secretase enzymes in the late endosomes.^71^ As endocytic trafficking is becoming increasingly recognized as a possible mechanism(s) of AD pathogenesis, examining the role of other endocytic trafficking regulators such as diacylglycerol kinase, Eps15 homology domain and molecules interacting with CasL-like1 (MICAL-L1) in both processing and release of APP via the exosomes can be developed as future areas of research that will provide valuable insights into the pathogenesis of AD.^76, 77, 78^ Interestingly, aggregated tau protein has also been found to be present in the exosomes in both the *in vitro* taupathy models as well as in the cerebrospinal fluids of early Alzheimer's patients.^68^ It is worth noting that microglia have an important role in spreading both A*β* and tau through the EVs.^79, 80^ The advent and widespread application of new imaging techniques such as super-resolution microscopy and quantitative methodology are also avenues that continue to contribute in our understanding of the molecular mechanism(s) involved in the processing of APP and tau and their roles in the pathogenesis of AD.^75, 81^

## Parkinson's Disease and EVs

Parkinson's disease (PD), clinically characterized by hypokinesia, rigidity and tremor, is the second most common neurodegenerative disorder. The pathological features of PD comprise of widespread degeneration of dopaminergic neurons and aggregation of Lewy bodies and cytoplasm inclusion bodies of *α*-synuclein.^82^ Interestingly, similar to the spread of pathogenic tau and A*β* proteins, EVs also have crucial roles in the aggregation and the spread of *α*-synuclein, thereby propagating disease pathogenesis in PD.^83, 84, 85^

Increased levels of *α*-synuclein have been reported in the exosomes isolated from both plasma and CSF of PD patients with a significant correlation observed between disease severity and plasma exosomal *α*-synuclein levels.^85^ It has been reported that spread of *α*-synuclein between neurons via the exosome route confers cytotoxicity to the recipient cells, leading to increased accumulation of Lewy body throughout the various brain regions.^84^ Although it is not well understood how this process is regulated, both Tsunemi *et al.*^86^ and Kong *et al.*^87^ suggest the role of P-type ATPase ion pump (PARK9/ATP13A2) in regulating both the biogenesis of exosomes, as well as secretion of exosomal *α*-synuclein. In case of juvenile-onset PD, that is attributed to loss-of-function mutations in PARK9, it was demonstrated that knocking down of PARK9 resulted in the inhibition of exosomal secretion of *α*-synuclein, and that, reciprocally, overexpression of PARK9 resulted in localization of PARK9 in MVBs and was associated with release of *α*-synuclein from the exosomes.^86, 87^

Another molecular mechanism regulating the exosomal release of *α*-synuclein is the autophagy-lysosomal pathway (ALP) that degrades the enclosed cargo in the lysosome.^88, 89, 90^ It can be speculated that exosomal release of *α*-synuclein is likely an adaptive response to insufficient autophagic activity needed for the elimination of *α*-synuclein.^89^ Using bafilomycin A1, a pharmacological inhibitor that disrupts ALP by blocking fusion between autophagosomes and lysosomes, Alvarez-Erviti *et al.*^91^ demonstrated that disruption of lysosomal functions resulted in enhanced exosomal release and uptake of *α*-synuclein in SH-SY5Y cells. In line with this, Poehler *et al.*^88^ also observed that ameliorating cytosolic accumulation of *α*-synuclein in bafilomycin A1-treated *α*-synuclein-transgenic mice, resulted in enhanced exosomal release of *α*-synuclein, which in turn, resulted in neuroinflammation and cellular damage. It is also worth noting that there is evidence suggesting that the gangliosides present in the exosomes can contribute to a catalytic environment leading to increased aggregation of *α*-synuclein.^83^

The role of glial cells in mediating neuroinflammatory responses leading to neurodegenerative disorders has been well documented.^92, 93, 94^ It is not surprising, therefore, that microglial/monocytes also have a role in the PD through the regulation of exosomal activities.^95^ For example, Chang *et al.*^95^ found that exosomes derived from *α*-synuclein-exposed BV-2 microglia contained high levels of MHC class II and membrane TNF-*α* and, that treatment of rat cortical neurons with these activated exosomes resulted in the neuronal apoptosis.

## Amyotrophic Lateral Sclerosis and EVs

Amyotrophic lateral sclerosis (ALS) is a progressive neurodegenerative disorder often accompanied with frontotemporal dementia.^96^ In the United States ALS affects ~3.9 cases per 100 000 individuals, with increased prevalence in persons aged 60–69 years.^97^ Loss of spinal cord motor neurons is the prominent pathological feature of ALS that manifests as muscle weakness and respiratory failure.^98, 99^ Several genes have been found to be associated with ALS including superoxide dismutase-1 protein (SOD1), RNA-binding protein fused in sarcoma and TAR DNA-binding protein 43 (TARDBP).^100, 101^

Similar to A*β*, mutations of SOD1 in ALS result in aggregation of intracellular misfolded SOD1 protein and its spread.^102^ Both mutant and wild-type SOD1 have been shown to be transmitted through both micropinocytosis of the released protein aggregates from dying cells as well as by uptake of SOD1 in the EVs by the recipient cells.^102^ Secretion of exosomal SOD1 has also been observed in a widely used *in vitro* model of ALS that of mouse motor neuron-like NSC-34 cells overexpressing the mutant human SOD1 (G93A).^103, 104^ Moreover, it has also been reported that mutant SOD1-containing exosomes released from the astrocytes exerted neurotoxicicty.^105^ Mechanisms by which SOD1 is packaged into the exosomes, however, remain elusive. Intriguingly, the expression of mutant SOD1 (G127X, G85R) has been reported on the surface of exosomes,^104^ whereas that of the wild-type SOD1 is in the lumen of the exosomes.^106, 107^ Upon uptake by recipient cells, mutant SOD1 proteins function as templates for further misfolding and aggregation of the natively folded counterparts.^102^ Studies by Grad *et al.*^104^ reported inhibition of the conversion of SOD1 into its misfolded form in the recipient cells via the treatment of exosomes with antibodies specific for mutant SOD1, indicating thereby the therapeutic potential of drug delivery by exosomes.

Similarly, TDP-43 aggregates have also implicated to be released in the EVs and to initiate the intracellular aggregation of TDP-43 in the recipient cells.^108^ Although TDP-43 aggregates are present in both microvesicles and exosomes, microvesicular TDP-43 has been shown to be the favored source for uptake by the recipient cells, leading in turn, to increased toxicity compared with the free TDP-43.^109^ In the same study it has also been suggested that TDP-43 could also spread trans-synaptically in both the anterograde and retrograde manner. In addition, it was also shown that neurons exposed to either TDP-43-containing media (derived from cultured cells) or to brain lysates from ALS patients did take up TDP-43.

## Prion Disease and EVs

Prion diseases are a spectrum of transmissible lethal neurodegenerative diseases characterized by cognitive impairment, motor dysfunction, spongiosis, astrogliosis and cerebral deposition of insoluble PRNP (prion protein).^110, 111^ In prion diseases, the native form of prion protein PRNP^C^ is converted into PRNP^SC^, a pathogenic form that is protease-resistant and prone to aggregation.^112^

PRNP has been identified in the EVs derived from both CSF^113^ and blood.^114^ Accumulating evidence further suggests a key role of EVs in both the pathogenesis and propagation of prion diseases.^115, 116, 117^ One of the initial studies has demonstrated that PRNP-expressing cell line (RK13) robustly secreted PRNP through exosomes.^118^ Vella *et al.*^119^ further showed that exosomes released from PRNP-infected neuronal cell line (GT1-7) also induced prion propagation in both neuronal as well as non-neuronal cells. Guo *et al.*^120^ have shown that inhibiting exosome release reduces the intercellular transmission of PRNP in different PRNP-expressing cell lines *in vitro*. Taking advantage of pharmacological inhibitors and genetic approaches, the same group also demonstrated that neutral sphingomyelinase pathway has a crucial role in regulating the packaging of PRNP into exosomes.^121^ Consistent with these *in vitro* findings, it has also been shown that EVs derived from plasma of mice infected with variant Creutzfeldt–Jakob disease (vCJD), one of the prion diseases, contains PRNP.^122^ Due to the fact that transmission of vCJD has also been clinically reported to be associated with blood transfusion,^123^ it is plausible to hypothesize that EVs might have a significant role in the transmission of prion diseases. Interestingly, upregulated miRNAs including let-7b, miR-146a, miR-103, miR-125a-5p and miR-342-3p were found to be present in the EVs isolated from human tissue samples affected with prion diseases,^124^ suggesting thereby that PRNP-infected EVs also mediate the pathogenesis of prion disease through their contents in addition to spreading of PRNP.

However, much still remains to be discovered about the role of exosomes in prion diseases. It has been demonstrated that different strains of PRNP are secreted differentially from RK13 cell line possibly through various disparate cellular mechanisms.^125^ A better understanding of the role of EVs in prion diseases is thus critical in dissecting mechanism(s) underlying disease pathogenesis while also providing insights for other prion-like diseases such as AD and PD.^126^

## Exosomes as Therapeutic Conduits

Exosomes via their ability to deliver specific cargo are critical for cellular communication and physiology. This property of exosomes can also be exploited as a treatment strategy to deliver specific therapeutic molecules to the diseased tissues. An elegant study by Zhuang *et al.*^127^ analyzed the potential for exosome therapeutics, through a noninvasive nasal delivery method, for the treatment of neuroinflammatory diseases. Herein the authors showed that exosome-mediated delivery of curcumin and an inhibitor of signal transducer and activator of transcription selectively and rapidly targeted the microglia, and significantly mitigated disease pathogenesis in three experimental models of neuroinflammation. This study highlights a novel and a noninvasive therapeutic approach for drug delivery into the CNS. A review by Andaloussi *et al.*^128^ covers the potential for exosomes as a delivery system for transporting siRNAs across biological barriers such as the blood–brain barrier. Exosomes-containing siRNAs could provide a means of targeted therapeutic delivery into areas that are difficult to traverse, such as the brain.^129, 130^ The ability to non-invasively deliver therapeutic exosomes to the CNS, with cell-specific targeting, could provide a potential therapeutic approach for the treatment and eradication of HIV-associated neurocognitive disorders by targeting the latent reservoirs in the CNS. Indeed, the application of exosomal delivery of natural HIV-defense molecules to host cells has been shown to establish HIV resistance to uninfected cells of *vif*-deficient HIV infection.^131^ Combining noninvasive therapies with siRNA targeting to HIV-1/HIV proteins could, thus, provide new therapeutic approaches to treat HIV-associated end-organ pathologies. Delivery of HIV gene-specific siRNA to infected cells could control virus infection and could be considered as adjunctive treatment(s) in combination with other therapeutic modalities. An elegant review by Vlassov *et al.*^132^ covering a broader view of exosome therapeutics, including their use against tumors, sets the stage for future work. Further studies on specific applications of exosome-mediated therapeutic delivery to specific cells are warranted in the field.

Intriguingly, assessment of the specific, signature contents of disease-specific exosomes could provide useful information for future development of exosome-based therapeutics. Indeed, exosomes from milk, but not plasma, have been shown to contain inhibitory factors for HIV-1 infection of monocyte-derived dendritic cells (MDDCs) and for the subsequent viral transfer to CD4 T cells through binding of MDDCs via DC-SIGN.^133^ In addition, there are several reports highlighting the potential for delivery of modified miRNAs and normal siRNAs to specific targets via the exosomes.^129, 130, 134, 135^ For more details please refer to the comprehensive review on the specific potential of targeting HIV and exosomal miRNAs as therapeutic approaches.^8, 136, 137^

Besides the targeting of exosomal contents, the exosome itself could be a potential therapeutic target. It is well documented that accumulation of reactive oxygen species underlies PD-associated neuroinflammation and neurotoxicity.^138^ Exosomes loaded with either antioxidant enzyme catalase or a plasmid DNA-encoding catalase have been used to reduce neuroinflammatory responses and exert neuroprotective effects.^139, 140^ Similarly, neurotropic factors that improve neuronal functioning can also be developed as potential therapeutic cargos for exosomal delivery. Along these lines Zhao *et al.* have demonstrated that delivery of macrophage exosomes containing glial cell line-derived neurotropic factor ameliorated neurodegeneration and neuroinflammation in PD mice.^141^

## Conclusions

In summary the exponential growth in our understanding of exosome biogenesis, composition, function and their use continues to provide new insights into the normal physiology as well as disease processes. These small vesicles are secreted by many cell types, including all of the CNS cells, and are a key component for cell–cell communication. They have crucial roles in various diseases including cancer metastasis. This review focuses mainly on the possible link between exosomes, HIV-1 pathogenesis and HIV-associated CNS disease. The ability to target exosomes involved in HIV-1 pathogenesis could provide a new means of controlling infection, which in turn, could help to curtail many of the commonly associated complications of the CNS. Further studies are needed concerning the application of exosome therapeutics, involving the use of these vesicles as a drug delivery conduits and as therapeutic targets themselves, in the context of battling HIV-1 infection and its associated end-organ pathologies.

## Figures and Tables

**Table 1:** Similarities and differences among HIV and EVs

	**HIV**	**Exosome**	**Microvesicle, Ectosome**	**Apoptotic body**
Size	90–160 nm	30–100 nm	100–1000 nm	50–5000 nm
Origin cell	Infected cell	Resting or activated cell	Activated or tumor cell	Apoptotic cell
Marker/Protein	Gag, Pol, gp120, Tat, Rev, Nef, Vpr, Vif and Vpu	CD63, CD9, Alix, Tsg101 and HSP70	Annexin V, flotillin-2, selectin, integrin and CD40 metalloproteinase	Annexin V, DNA and histones
Budding mechanism	Exocytosis from MVB	Exocytosis from MVB	Budding directly from a plasma membrane (outward budding)	Programmed cell death (blebbing)
Involved components	ESCRT components, Tsg101, Alix and HIV-1 Gag	nSMAse2, ceramide, ESCRT components and ARRDC1, Tsg101, Alix and tetraspanin	aSMAse, ceramide, ESCRT components, Tsg101, Vps4 and tetraspanin	Caspase-3, rho-associated coiled-coil-forming kinase I and actin polymerization
Entry mechanism	HIV viral envelope, gp120 and gp41, binding CD4	Direct fusion, phagocytosis and RME (Figure 1).	Direct fusion, phagocytosis and RME (Figure 1).	Phagocytosis
Occur position	Plasma membrane and MVB	Plasma membrane and MVB	Plasma membrane	Plasma membrane

## Abbreviations

CNS: central nervous system
MVBs: multi-vesicular bodies
EVs: extracellular vesicles
ILVs: intraluminal vesicles
EBV: Epstein–Barr virus
RME: receptor-mediated endocytosis
ESCRT: endosomal sorting complexes required for transport
ARF6: ADP ribosylation factor 6
PLD2: phospholipase D2
miRNAs: microRNAs
PS: phosphatidylserine
MDMs: monocyte-derived macrophages
AD: Alzheimer disease
A*β*: amyloid beta
APP: amyloid precursor protein
APP-CTFs: C-terminal fragments of APP
PD: Parkinson's disease
ALP: autophagy-lysosomal pathway
ALS: amyotrophic lateral sclerosis
SOD1: superoxide dismutase-1 protein
TARDBP: TAR DNA-binding protein
vCJD: variant Creutzfeldt–Jakob disease
MDDCs: monocyte-derived dendritic cells

## References

[bib1] Trams EG, Lauter CJ, Salem N Jr., Heine U. Exfoliation of membrane ecto-enzymes in the form of micro-vesicles. Biochim Biophys Acta 1981; 645: 63–70. 6266476 10.1016/0005-2736(81)90512-5

[bib2] Pan BT, Teng K, Wu C, Adam M, Johnstone RM. Electron microscopic evidence for externalization of the transferrin receptor in vesicular form in sheep reticulocytes. J Cell Biol 1985; 101: 942–948. 2993317 10.1083/jcb.101.3.942 PMC2113705

[bib3] Johnstone RM, Adam M, Hammond JR, Orr L, Turbide C. Vesicle formation during reticulocyte maturation. Association of plasma membrane activities with released vesicles (exosomes). J Biol Chem 1987; 262: 9412–9420. 3597417

[bib4] Gupta A, Pulliam L. Exosomes as mediators of neuroinflammation. J Neuroinflammation 2014; 11: 68. 24694258 10.1186/1742-2094-11-68 PMC3994210

[bib5] Trajkovic K, Hsu C, Chiantia S, Rajendran L, Wenzel D, Wieland F et al. Ceramide triggers budding of exosome vesicles into multivesicular endosomes. Science 2008; 319: 1244–1247. 18309083 10.1126/science.1153124

[bib6] Ghossoub R, Lembo F, Rubio A, Gaillard CB, Bouchet J, Vitale N et al. Syntenin-ALIX exosome biogenesis and budding into multivesicular bodies are controlled by ARF6 and PLD2. Nat Commun 2014; 5: 3477. 24637612 10.1038/ncomms4477

[bib7] Thery C, Zitvogel L, Amigorena S. Exosomes: composition, biogenesis and function. Nat Rev Immunol 2002; 2: 569–579. 12154376 10.1038/nri855

[bib8] Hu G, Drescher KM, Chen XM. Exosomal miRNAs: biological properties and therapeutic potential. Front Genet 2012; 3: 56. 22529849 10.3389/fgene.2012.00056 PMC3330238

[bib9] Schorey JS, Bhatnagar S. Exosome function: from tumor immunology to pathogen biology. Traffic 2008; 9: 871–881. 18331451 10.1111/j.1600-0854.2008.00734.x PMC3636814

[bib10] Gonzales PA, Pisitkun T, Hoffert JD, Tchapyjnikov D, Star RA, Kleta R et al. Large-scale proteomics and phosphoproteomics of urinary exosomes. J Am Soc Nephrol 2009; 20: 363–379. 19056867 10.1681/ASN.2008040406 PMC2637050

[bib11] Valadi H, Ekstrom K, Bossios A, Sjostrand M, Lee JJ, Lotvall JO. Exosome-mediated transfer of mRNAs and microRNAs is a novel mechanism of genetic exchange between cells. Nat Cell Biol 2007; 9: 654–659. 17486113 10.1038/ncb1596

[bib12] Bartel DP. MicroRNAs: target recognition and regulatory functions. Cell 2009; 136: 215–233. 19167326 10.1016/j.cell.2009.01.002 PMC3794896

[bib13] Mathivanan S, Fahner CJ, Reid GE, Simpson RJ. ExoCarta 2012: database of exosomal proteins, RNA and lipids. Nucleic Acids Res 2012; 40: D1241–D1244. 21989406 10.1093/nar/gkr828 PMC3245025

[bib14] Faure J, Lachenal G, Court M, Hirrlinger J, Chatellard-Causse C, Blot B et al. Exosomes are released by cultured cortical neurons. Mol Cell Neurosci 2006; 31: 642–648. 16446100 10.1016/j.mcn.2005.12.003

[bib15] Thery C, Ostrowski M, Segura E. Membrane vesicles as conveyors of immune responses. Nat Rev Immunol 2009; 9: 581–593. 19498381 10.1038/nri2567

[bib16] Meckes DG Jr., Shair KH, Marquitz AR, Kung CP, Edwards RH, Raab-Traub N. Human tumor virus utilizes exosomes for intercellular communication. Proc Natl Acad Sci USA 2010; 107: 20370–20375. 21059916 10.1073/pnas.1014194107 PMC2996715

[bib17] Witwer KW, Buzas EI, Bemis LT, Bora A, Lasser C, Lotvall J et al. Standardization of sample collection, isolation and analysis methods in extracellular vesicle research. J Extracell Vesicles 2013; 2. 10.3402/jev.v2i0.20360 PMC3760646 24009894

[bib18] Keller S, Konig AK, Marme F, Runz S, Wolterink S, Koensgen D et al. Systemic presence and tumor-growth promoting effect of ovarian carcinoma released exosomes. Cancer Lett 2009; 278: 73–81. 19188015 10.1016/j.canlet.2008.12.028

[bib19] Cocucci E, Racchetti G, Meldolesi J. Shedding microvesicles: artefacts no more. Trends Cell Biol 2009; 19: 43–51. 19144520 10.1016/j.tcb.2008.11.003

[bib20] Raposo G, Stoorvogel W. Extracellular vesicles: exosomes, microvesicles, and friends. J Cell Biol 2013; 200: 373–383. 23420871 10.1083/jcb.201211138 PMC3575529

[bib21] Chertova E, Chertov O, Coren LV, Roser JD, Trubey CM, Bess JW Jr. et al. Proteomic and biochemical analysis of purified human immunodeficiency virus type 1 produced from infected monocyte-derived macrophages. J Virol 2006; 80: 9039–9052. 16940516 10.1128/JVI.01013-06 PMC1563931

[bib22] Miyanishi M, Tada K, Koike M, Uchiyama Y, Kitamura T, Nagata S. Identification of Tim4 as a phosphatidylserine receptor. Nature 2007; 450: 435–439. 17960135 10.1038/nature06307

[bib23] Zakharova L, Svetlova M, Fomina AF. T cell exosomes induce cholesterol accumulation in human monocytes via phosphatidylserine receptor. J Cell Physiol 2007; 212: 174–181. 17299798 10.1002/jcp.21013

[bib24] Escrevente C, Keller S, Altevogt P, Costa J. Interaction and uptake of exosomes by ovarian cancer cells. BMC Cancer 2011; 11: 108. 21439085 10.1186/1471-2407-11-108 PMC3072949

[bib25] Skokos D, Botros HG, Demeure C, Morin J, Peronet R, Birkenmeier G et al. Mast cell-derived exosomes induce phenotypic and functional maturation of dendritic cells and elicit specific immune responses *in vivo*. J Immunol 2003; 170: 3037–3045. 12626558 10.4049/jimmunol.170.6.3037

[bib26] Kooijmans SA, Vader P, van Dommelen SM, van Solinge WW, Schiffelers RM. Exosome mimetics: a novel class of drug delivery systems. Int J Nanomedicine 2012; 7: 1525–1541. 22619510 10.2147/IJN.S29661 PMC3356169

[bib27] Feng D, Zhao WL, Ye YY, Bai XC, Liu RQ, Chang LF et al. Cellular internalization of exosomes occurs through phagocytosis. Traffic 2010; 11: 675–687. 20136776 10.1111/j.1600-0854.2010.01041.x

[bib28] Fitzner D, Schnaars M, van Rossum D, Krishnamoorthy G, Dibaj P, Bakhti M et al. Selective transfer of exosomes from oligodendrocytes to microglia by macropinocytosis. J Cell Sci 2011; 124: 447–458. 21242314 10.1242/jcs.074088

[bib29] Stoorvogel W, Kleijmeer MJ, Geuze HJ, Raposo G. The biogenesis and functions of exosomes. Traffic 2002; 3: 321–330. 11967126 10.1034/j.1600-0854.2002.30502.x

[bib30] Nguyen DG, Booth A, Gould SJ, Hildreth JE. Evidence that HIV budding in primary macrophages occurs through the exosome release pathway. J Biol Chem 2003; 278: 52347–52354. 14561735 10.1074/jbc.M309009200

[bib31] Bieniasz PD. The cell biology of HIV-1 virion genesis. Cell Host Microbe 2009; 5: 550–558. 19527882 10.1016/j.chom.2009.05.015 PMC3736989

[bib32] Nabhan JF, Hu R, Oh RS, Cohen SN, Lu Q. Formation and release of arrestin domain-containing protein 1-mediated microvesicles (ARMMs) at plasma membrane by recruitment of TSG101 protein. Proc Natl Acad Sci USA 2012; 109: 4146–4151. 22315426 10.1073/pnas.1200448109 PMC3306724

[bib33] van Dongen HM, Masoumi N, Witwer KW, Pegtel DM. Extracellular vesicles exploit viral entry routes for cargo delivery. Microbiol Mol Biol Rev 2016; 80: 369–386. 26935137 10.1128/MMBR.00063-15 PMC4867369

[bib34] Park IW, He JJ. HIV-1 is budded from CD4+ T lymphocytes independently of exosomes. Virol J 2010; 7: 234. 20846372 10.1186/1743-422X-7-234 PMC2945958

[bib35] Garrus JE, von Schwedler UK, Pornillos OW, Morham SG, Zavitz KH, Wang HE et al. Tsg101 and the vacuolar protein sorting pathway are essential for HIV-1 budding. Cell 2001; 107: 55–65. 11595185 10.1016/s0092-8674(01)00506-2

[bib36] Morita E, Sundquist WI. Retrovirus budding. Annu Rev Cell Dev Biol 2004; 20: 395–425. 15473846 10.1146/annurev.cellbio.20.010403.102350

[bib37] Booth AM, Fang Y, Fallon JK, Yang JM, Hildreth JE, Gould SJ. Exosomes and HIV Gag bud from endosome-like domains of the T cell plasma membrane. J Cell Biol 2006; 172: 923–935. 16533950 10.1083/jcb.200508014 PMC2063735

[bib38] Gottlinger HG, Dorfman T, Sodroski JG, Haseltine WA. Effect of mutations affecting the p6 gag protein on human immunodeficiency virus particle release. Proc Natl Acad Sci USA 1991; 88: 3195–3199. 2014240 10.1073/pnas.88.8.3195 PMC51412

[bib39] Gan X, Gould SJ. HIV Pol inhibits HIV budding and mediates the severe budding defect of Gag-Pol. PLoS One 2012; 7: e29421. 22235295 10.1371/journal.pone.0029421 PMC3250436

[bib40] Kadiu I, Narayanasamy P, Dash PK, Zhang W, Gendelman HE. Biochemical and biologic characterization of exosomes and microvesicles as facilitators of HIV-1 infection in macrophages. J Immunol 2012; 189: 744–754. 22711894 10.4049/jimmunol.1102244 PMC3786185

[bib41] Li M, Aliotta JM, Asara JM, Tucker L, Quesenberry P, Lally M et al. Quantitative proteomic analysis of exosomes from HIV-1-infected lymphocytic cells. Proteomics 2012; 12: 2203–2211. 22807456 10.1002/pmic.201100376 PMC3815571

[bib42] Jaworski E, Narayanan A, Van Duyne R, Shabbeer-Meyering S, Iordanskiy S, Saifuddin M et al. Human T-lymphotropic virus type 1-infected cells secrete exosomes that contain Tax protein. J Biol Chem 2014; 289: 22284–22305. 24939845 10.1074/jbc.M114.549659 PMC4139239

[bib43] Columba Cabezas S, Federico M. Sequences within RNA coding for HIV-1 Gag p17 are efficiently targeted to exosomes. Cell Microbiol 2013; 15: 412–429. 23072732 10.1111/cmi.12046

[bib44] Narayanan A, Iordanskiy S, Das R, Van Duyne R, Santos S, Jaworski E et al. Exosomes derived from HIV-1-infected cells contain trans-activation response element RNA. J Biol Chem 2013; 288: 20014–20033. 23661700 10.1074/jbc.M112.438895 PMC3707700

[bib45] Sampey GC, Saifuddin M, Schwab A, Barclay R, Punya S, Chung MC et al. Exosomes from HIV-1-infected cells stimulate production of pro-inflammatory cytokines through trans-activating response (TAR) RNA. J Biol Chem 2016; 291: 1251–1266. 26553869 10.1074/jbc.M115.662171 PMC4714213

[bib46] Hu G, Yao H, Chaudhuri AD, Duan M, Yelamanchili SV, Wen H et al. Exosome-mediated shuttling of microRNA-29 regulates HIV Tat and morphine-mediated neuronal dysfunction. Cell Death Dis 2012; 3: e381. 22932723 10.1038/cddis.2012.114 PMC3434655

[bib47] Yelamanchili SV, Lamberty BG, Rennard DA, Morsey BM, Hochfelder CG, Meays BM et al. Correction: MiR-21 in extracellular vesicles leads to neurotoxicity via TLR7 signaling in SIV neurological disease. PLoS Pathog 2015; 11: e1005131. 26327533 10.1371/journal.ppat.1005131 PMC4556636

[bib48] Roth WW, Huang MB, Addae Konadu K, Powell MD, Bond VC. Micro RNA in exosomes from HIV-infected macrophages. Int J Environ Res Public Health 2015; 13: 32. 10.3390/ijerph13010032 PMC4730423 26703692

[bib49] Ali SA, Huang MB, Campbell PE, Roth WW, Campbell T, Khan M et al. Genetic characterization of HIV type 1 Nef-induced vesicle secretion. AIDS Res Hum Retroviruses 2010; 26: 173–192. 20156100 10.1089/aid.2009.0068 PMC2835390

[bib50] Campbell TD, Khan M, Huang MB, Bond VC, Powell MD. HIV-1 Nef protein is secreted into vesicles that can fuse with target cells and virions. Ethn Dis 2008; 18: S2–14–19. PMC3418053 18646314

[bib51] Lenassi M, Cagney G, Liao M, Vaupotic T, Bartholomeeusen K, Cheng Y et al. HIV Nef is secreted in exosomes and triggers apoptosis in bystander CD4+ T cells. Traffic 2010; 11: 110–122. 19912576 10.1111/j.1600-0854.2009.01006.x PMC2796297

[bib52] Muratori C, Cavallin LE, Kratzel K, Tinari A, De Milito A, Fais S et al. Massive secretion by T cells is caused by HIV Nef in infected cells and by Nef transfer to bystander cells. Cell Host Microbe 2009; 6: 218–230. 19748464 10.1016/j.chom.2009.06.009

[bib53] Raymond AD, Campbell-Sims TC, Khan M, Lang M, Huang MB, Bond VC et al. HIV Type 1 Nef is released from infected cells in CD45(+) microvesicles and is present in the plasma of HIV-infected individuals. AIDS Res Hum Retroviruses 2011; 27: 167–178. 20964480 10.1089/aid.2009.0170 PMC3064529

[bib54] Shelton MN, Huang MB, Ali SA, Powell MD, Bond VC. Secretion modification region-derived peptide disrupts HIV-1 Nef's interaction with mortalin and blocks virus and Nef exosome release. J Virol 2012; 86: 406–419. 22013042 10.1128/JVI.05720-11 PMC3255900

[bib55] Rahimian P, He JJ. Exosome-associated release, uptake, and neurotoxicity of HIV-1 Tat protein. J Neurovirol 2016; (e-pub ahead of print). 10.1007/s13365-016-0451-6 PMC5690550 27173397

[bib56] Konadu KA, Chu J, Huang MB, Amancha PK, Armstrong W, Powell MD et al. Association of cytokines with exosomes in the plasma of HIV-1-seropositive individuals. J Infect Dis 2015; 211: 1712–1716. 25512626 10.1093/infdis/jiu676 PMC4447830

[bib57] Schwab A, Meyering SS, Lepene B, Iordanskiy S, van Hoek ML, Hakami RM et al. Extracellular vesicles from infected cells: potential for direct pathogenesis. Front Microbiol 2015; 6: 1132. 26539170 10.3389/fmicb.2015.01132 PMC4611157

[bib58] Jaworski E, Saifuddin M, Sampey G, Shafagati N, Van Duyne R, Iordanskiy S et al. The use of Nanotrap particles technology in capturing HIV-1 virions and viral proteins from infected cells. PLoS One 2014; 9: e96778. 24820173 10.1371/journal.pone.0096778 PMC4018389

[bib59] Arenaccio C, Chiozzini C, Columba-Cabezas S, Manfredi F, Affabris E, Baur A et al. Exosomes from human immunodeficiency virus type-1 (HIV-1)-infected cells license quiescent CD4+ T lymphocytes to replicate HIV-1 through a Nef- and ADAM17-dependent mechanism. J Virol 2014; 88: 11529–11539. 25056899 10.1128/JVI.01712-14 PMC4178784

[bib60] Arenaccio C, Chiozzini C, Columba-Cabezas S, Manfredi F, Federico M. Cell activation and HIV-1 replication in unstimulated CD4+ T lymphocytes ingesting exosomes from cells expressing defective HIV-1. Retrovirology 2014; 11: 46. 24924541 10.1186/1742-4690-11-46 PMC4229896

[bib61] Aqil M, Naqvi AR, Mallik S, Bandyopadhyay S, Maulik U, Jameel S. The HIV Nef protein modulates cellular and exosomal miRNA profiles in human monocytic cells. J Extracell Vesicles 2014; 3. 10.3402/jev.v3.23129 PMC3967016 24678387

[bib62] Gulzar N, Copeland KF. CD8+ T-cells: function and response to HIV infection. Curr HIV Res 2004; 2: 23–37. 15053338 10.2174/1570162043485077

[bib63] Tumne A, Prasad VS, Chen Y, Stolz DB, Saha K, Ratner DM et al. Noncytotoxic suppression of human immunodeficiency virus type 1 transcription by exosomes secreted from CD8+ T cells. J Virol 2009; 83: 4354–4364. 19193788 10.1128/JVI.02629-08 PMC2668436

[bib64] Prince M, Wimo A, Guerchet M, Ali G, Wu Y, Prina M. World Alzheimer Report 2015. The global impact of dementia. An analysis of prevalence, incidence, cost and trends. Alzheimers Dis Int 2015.

[bib65] Selkoe DJ. Alzheimer's disease: genes, proteins, and therapy. Physiol Rev 2001; 81: 741–766. 11274343 10.1152/physrev.2001.81.2.741

[bib66] Goetzl EJ, Boxer A, Schwartz JB, Abner EL, Petersen RC, Miller BL et al. Low neural exosomal levels of cellular survival factors in Alzheimer's disease. Ann Clin Transl Neurol 2015; 2: 769–773. 26273689 10.1002/acn3.211 PMC4531059

[bib67] Fiandaca MS, Kapogiannis D, Mapstone M, Boxer A, Eitan E, Schwartz JB et al. Identification of preclinical Alzheimer's disease by a profile of pathogenic proteins in neurally derived blood exosomes: a case-control study. Alzheimers Dement 2015; 11: 600–607 e601. 25130657 10.1016/j.jalz.2014.06.008 PMC4329112

[bib68] Saman S, Kim W, Raya M, Visnick Y, Miro S, Saman S et al. Exosome-associated tau is secreted in tauopathy models and is selectively phosphorylated in cerebrospinal fluid in early Alzheimer disease. J Biol Chem 2012; 287: 3842–3849. 22057275 10.1074/jbc.M111.277061 PMC3281682

[bib69] Kokubo H, Saido TC, Iwata N, Helms JB, Shinohara R, Yamaguchi H. Part of membrane-bound Abeta exists in rafts within senile plaques in Tg2576 mouse brain. Neurobiol Aging 2005; 26: 409–418. 15653169 10.1016/j.neurobiolaging.2004.04.008

[bib70] Rajendran L, Honsho M, Zahn TR, Keller P, Geiger KD, Verkade P et al. Alzheimer's disease beta-amyloid peptides are released in association with exosomes. Proc Natl Acad Sci USA 2006; 103: 11172–11177. 16837572 10.1073/pnas.0603838103 PMC1544060

[bib71] Sullivan CP, Jay AG, Stack EC, Pakaluk M, Wadlinger E, Fine RE et al. Retromer disruption promotes amyloidogenic APP processing. Neurobiol Dis 2011; 43: 338–345. 21515373 10.1016/j.nbd.2011.04.002 PMC3114192

[bib72] Sharples RA, Vella LJ, Nisbet RM, Naylor R, Perez K, Barnham KJ et al. Inhibition of gamma-secretase causes increased secretion of amyloid precursor protein C-terminal fragments in association with exosomes. FASEB J 2008; 22: 1469–1478. 18171695 10.1096/fj.07-9357com

[bib73] Cai Y, Arikkath J, Yang L, Guo ML, Periyasamy P, Buch S. Interplay of endoplasmic reticulum stress and autophagy in neurodegenerative disorders. Autophagy 2016; 12: 225–244. 26902584 10.1080/15548627.2015.1121360 PMC4835965

[bib74] Zhang M, Haapasalo A, Kim DY, Ingano LA, Pettingell WH, Kovacs DM. Presenilin/gamma-secretase activity regulates protein clearance from the endocytic recycling compartment. FASEB J 2006; 20: 1176–1178. 16645046 10.1096/fj.05-5531fje

[bib75] Xie S, Bahl K, Reinecke JB, Hammond GR, Naslavsky N, Caplan S. The endocytic recycling compartment maintains cargo segregation acquired upon exit from the sorting endosome. Mol Biol Cell 2016; 27: 108–126. 26510502 10.1091/mbc.E15-07-0514 PMC4694750

[bib76] Xie S, Naslavsky N, Caplan S. Diacylglycerol kinases in membrane trafficking. Cell Logist 2015; 5: e1078431. 27057419 10.1080/21592799.2015.1078431 PMC4820814

[bib77] Xie S, Naslavsky N, Caplan S. Diacylglycerol kinase alpha regulates tubular recycling endosome biogenesis and major histocompatibility complex class I recycling. J Biol Chem 2014; 289: 31914–31926. 25248744 10.1074/jbc.M114.594291 PMC4231670

[bib78] Cai B, Xie S, Caplan S, Naslavsky N. GRAF1 forms a complex with MICAL-L1 and EHD1 to cooperate in tubular recycling endosome vesiculation. Front Cell Dev Biol 2014; 2: 22. 25364729 10.3389/fcell.2014.00022 PMC4214196

[bib79] Joshi P, Turola E, Ruiz A, Bergami A, Libera DD, Benussi L et al. Microglia convert aggregated amyloid-beta into neurotoxic forms through the shedding of microvesicles. Cell Death Differ 2014; 21: 582–593. 24336048 10.1038/cdd.2013.180 PMC3950321

[bib80] Asai H, Ikezu S, Tsunoda S, Medalla M, Luebke J, Haydar T et al. Depletion of microglia and inhibition of exosome synthesis halt tau propagation. Nat Neurosci 2015; 18: 1584–1593. 26436904 10.1038/nn.4132 PMC4694577

[bib81] Reineke JB, Xie S, Naslavsky N, Caplan S. Qualitative and quantitative analysis of endocytic recycling. Methods Cell Biol 2015; 130: 139–155. 26360033 10.1016/bs.mcb.2015.04.002 PMC13274225

[bib82] Pollanen MS, Dickson DW, Bergeron C. Pathology and biology of the Lewy body. J Neuropathol Exp Neurol 1993; 52: 183–191. 7684074 10.1097/00005072-199305000-00001

[bib83] Grey M, Dunning CJ, Gaspar R, Grey C, Brundin P, Sparr E et al. Acceleration of alpha-synuclein aggregation by exosomes. J Biol Chem 2015; 290: 2969–2982. 25425650 10.1074/jbc.M114.585703 PMC4317028

[bib84] Gallegos S, Pacheco C, Peters C, Opazo CM, Aguayo LG. Features of alpha-synuclein that could explain the progression and irreversibility of Parkinson's disease. Front Neurosci 2015; 9: 59. 25805964 10.3389/fnins.2015.00059 PMC4353246

[bib85] Shi M, Liu C, Cook TJ, Bullock KM, Zhao Y, Ginghina C et al. Plasma exosomal alpha-synuclein is likely CNS-derived and increased in Parkinson's disease. Acta Neuropathol 2014; 128: 639–650. 24997849 10.1007/s00401-014-1314-y PMC4201967

[bib86] Tsunemi T, Hamada K, Krainc D. ATP13A2/PARK9 regulates secretion of exosomes and alpha-synuclein. J Neurosci 2014; 34: 15281–15287. 25392495 10.1523/JNEUROSCI.1629-14.2014 PMC4228131

[bib87] Kong SM, Chan BK, Park JS, Hill KJ, Aitken JB, Cottle L et al. Parkinson's disease-linked human PARK9/ATP13A2 maintains zinc homeostasis and promotes alpha-Synuclein externalization via exosomes. Hum Mol Genet 2014; 23: 2816–2833. 24603074 10.1093/hmg/ddu099

[bib88] Poehler AM, Xiang W, Spitzer P, May VE, Meixner H, Rockenstein E et al. Autophagy modulates SNCA/alpha-synuclein release, thereby generating a hostile microenvironment. Autophagy 2014; 10: 2171–2192. 25484190 10.4161/auto.36436 PMC4502760

[bib89] Danzer KM, Kranich LR, Ruf WP, Cagsal-Getkin O, Winslow AR, Zhu L et al. Exosomal cell-to-cell transmission of alpha synuclein oligomers. Mol Neurodegener 2012; 7: 42. 22920859 10.1186/1750-1326-7-42 PMC3483256

[bib90] Cai B, Xie S, Liu F, Simone LC, Caplan S, Qin X et al. Rapid degradation of the complement regulator, CD59, by a novel inhibitor. J Biol Chem 2014; 289: 12109–12125. 24616098 10.1074/jbc.M113.547083 PMC4002116

[bib91] Alvarez-Erviti L, Seow Y, Schapira AH, Gardiner C, Sargent IL, Wood MJ et al. Lysosomal dysfunction increases exosome-mediated alpha-synuclein release and transmission. Neurobiol Dis 2011; 42: 360–367. 21303699 10.1016/j.nbd.2011.01.029 PMC3107939

[bib92] Yang L, Yao H, Chen X, Cai Y, Callen S, Buch S. Role of sigma receptor in cocaine-mediated induction of glial fibrillary acidic protein: implications for HAND. Mol Neurobiol 2016; 53: 1329–1342. 25631712 10.1007/s12035-015-9094-5 PMC4519438

[bib93] Duan M, Yao H, Cai Y, Liao K, Seth P, Buch S. HIV-1 Tat disrupts CX3CL1-CX3CR1 axis in microglia via the NF-kappaBYY1 pathway. Curr HIV Res 2014; 12: 189–200. 24862326 10.2174/1570162x12666140526123119 PMC5003019

[bib94] Yao H, Bethel-Brown C, Yang L, Cai Y, Kanmogne M, Mudgapalli V et al. Signal transduction in HIV protein-treated astrocytes. Curr Sig Transduct Ther 2012; 7: 28–34.

[bib95] Chang C, Lang H, Geng N, Wang J, Li N, Wang X. Exosomes of BV-2 cells induced by alpha-synuclein: important mediator of neurodegeneration in PD. Neurosci Lett 2013; 548: 190–195. 23792198 10.1016/j.neulet.2013.06.009

[bib96] Ferrari R, Kapogiannis D, Huey ED, Momeni P. FTD and ALS: a tale of two diseases. Curr Alzheimer Res 2011; 8: 273–294. 21222600 10.2174/156720511795563700 PMC3801195

[bib97] Mehta P, Antao V, Kaye W, Sanchez M, Williamson D, Bryan L et al. Prevalence of amyotrophic lateral sclerosis - United States, 2010-2011. MMWR Suppl 2014; 63: 1–14. 25054277

[bib98] Boillee S, Vande Velde C, Cleveland DW. ALS: a disease of motor neurons and their nonneuronal neighbors. Neuron 2006; 52: 39–59. 17015226 10.1016/j.neuron.2006.09.018

[bib99] Robberecht W, Philips T. The changing scene of amyotrophic lateral sclerosis. Nat Rev Neurosci 2013; 14: 248–264. 23463272 10.1038/nrn3430

[bib100] Millecamps S, Salachas F, Cazeneuve C, Gordon P, Bricka B, Camuzat A et al. SOD1, ANG, VAPB, TARDBP, and FUS mutations in familial amyotrophic lateral sclerosis: genotype-phenotype correlations. J Med Genet 2010; 47: 554–560. 20577002 10.1136/jmg.2010.077180

[bib101] Daoud H, Valdmanis PN, Kabashi E, Dion P, Dupre N, Camu W et al. Contribution of TARDBP mutations to sporadic amyotrophic lateral sclerosis. J Med Genet 2009; 46: 112–114. 18931000 10.1136/jmg.2008.062463

[bib102] Silverman JM, Fernando SM, Grad LI, Hill AF, Turner BJ, Yerbury JJ et al. Disease Mechanisms in ALS: misfolded SOD1 transferred through exosome-dependent and exosome-independent pathways. Cell Mol Neurobiol 2016; 36: 377–381. 26908139 10.1007/s10571-015-0294-3 PMC11482315

[bib103] Gomes C, Keller S, Altevogt P, Costa J. Evidence for secretion of Cu, Zn superoxide dismutase via exosomes from a cell model of amyotrophic lateral sclerosis. Neurosci Lett 2007; 428: 43–46. 17942226 10.1016/j.neulet.2007.09.024

[bib104] Grad LI, Yerbury JJ, Turner BJ, Guest WC, Pokrishevsky E, O'Neill MA et al. Intercellular propagated misfolding of wild-type Cu/Zn superoxide dismutase occurs via exosome-dependent and -independent mechanisms. Proc Natl Acad Sci USA 2014; 111: 3620–3625. 24550511 10.1073/pnas.1312245111 PMC3948312

[bib105] Basso M, Pozzi S, Tortarolo M, Fiordaliso F, Bisighini C, Pasetto L et al. Mutant copper-zinc superoxide dismutase (SOD1) induces protein secretion pathway alterations and exosome release in astrocytes: implications for disease spreading and motor neuron pathology in amyotrophic lateral sclerosis. J Biol Chem 2013; 288: 15699–15711. 23592792 10.1074/jbc.M112.425066 PMC3668729

[bib106] Yuyama K, Igarashi Y. Physiological and pathological roles of exosomes in the nervous system. Biomol Concepts 2016; 7: 53–68. 26812803 10.1515/bmc-2015-0033

[bib107] Kim DK, Kang B, Kim OY, Choi DS, Lee J, Kim SR et al. EVpedia: an integrated database of high-throughput data for systemic analyses of extracellular vesicles. J Extracell Vesicles 2013; 2. 10.3402/jev.v2i0.20384 PMC3760654 24009897

[bib108] Nonaka T, Masuda-Suzukake M, Arai T, Hasegawa Y, Akatsu H, Obi T et al. Prion-like properties of pathological TDP-43 aggregates from diseased brains. Cell Rep 2013; 4: 124–134. 23831027 10.1016/j.celrep.2013.06.007

[bib109] Feiler MS, Strobel B, Freischmidt A, Helferich AM, Kappel J, Brewer BM et al. TDP-43 is intercellularly transmitted across axon terminals. J Cell Biol 2015; 211: 897–911. 26598621 10.1083/jcb.201504057 PMC4657165

[bib110] Ugalde CL, Finkelstein DI, Lawson VA, Hill AF. Pathogenic mechanisms of prion protein, amyloid-beta and alpha-synuclein misfolding: the prion concept and neurotoxicity of protein oligomers. J Neurochem 2016; 139: 162–180. 27529376 10.1111/jnc.13772

[bib111] Tagliapietra M, Zanusso G, Fiorini M, Bonetto N, Zarantonello G, Zambon A et al. Accuracy of diagnostic criteria for sporadic Creutzfeldt-Jakob disease among rapidly progressive dementia. J Alzheimers Dis 2013; 34: 231–238. 23207489 10.3233/JAD-121873

[bib112] Hetz CA, Soto C. Stressing out the ER: a role of the unfolded protein response in prion-related disorders. Curr Mol Med 2006; 6: 37–43. 16472111 10.2174/156652406775574578 PMC2838391

[bib113] Vella LJ, Greenwood DL, Cappai R, Scheerlinck JP, Hill AF. Enrichment of prion protein in exosomes derived from ovine cerebral spinal fluid. Vet Immunol Immunopathol 2008; 124: 385–393. 18501435 10.1016/j.vetimm.2008.04.002

[bib114] Robertson C, Booth SA, Beniac DR, Coulthart MB, Booth TF, McNicol A. Cellular prion protein is released on exosomes from activated platelets. Blood 2006; 107: 3907–3911. 16434486 10.1182/blood-2005-02-0802

[bib115] Basso M, Bonetto V. Extracellular vesicles and a novel form of communication in the brain. Front Neurosci 2016; 10: 127. 27065789 10.3389/fnins.2016.00127 PMC4814526

[bib116] Vella LJ, Hill AF, Cheng L. Focus on extracellular vesicles: exosomes and their role in protein trafficking and biomarker potential in Alzheimer's and Parkinson's disease. Int J Mol Sci 2016; 17: 173. 26861304 10.3390/ijms17020173 PMC4783907

[bib117] Coleman BM, Hill AF. Extracellular vesicles—their role in the packaging and spread of misfolded proteins associated with neurodegenerative diseases. Semin Cell Dev Biol 2015; 40: 89–96. 25704308 10.1016/j.semcdb.2015.02.007

[bib118] Fevrier B, Vilette D, Archer F, Loew D, Faigle W, Vidal M et al. Cells release prions in association with exosomes. Proc Natl Acad Sci USA 2004; 101: 9683–9688. 15210972 10.1073/pnas.0308413101 PMC470735

[bib119] Vella LJ, Sharples RA, Lawson VA, Masters CL, Cappai R, Hill AF. Packaging of prions into exosomes is associated with a novel pathway of PrP processing. J Pathol 2007; 211: 582–590. 17334982 10.1002/path.2145

[bib120] Guo BB, Bellingham SA, Hill AF. Stimulating the release of exosomes increases the intercellular transfer of prions. J Biol Chem 2016; 291: 5128–5137. 26769968 10.1074/jbc.M115.684258 PMC4777847

[bib121] Guo BB, Bellingham SA, Hill AF. The neutral sphingomyelinase pathway regulates packaging of the prion protein into exosomes. J Biol Chem 2015; 290: 3455–3467. 25505180 10.1074/jbc.M114.605253 PMC4319014

[bib122] Saa P, Yakovleva O, de Castro J, Vasilyeva I, De Paoli SH, Simak J et al. First demonstration of transmissible spongiform encephalopathy-associated prion protein (PrPTSE) in extracellular vesicles from plasma of mice infected with mouse-adapted variant Creutzfeldt-Jakob disease by *in vitro* amplification. J Biol Chem 2014; 289: 29247–29260. 25157106 10.1074/jbc.M114.589564 PMC4200276

[bib123] Wroe SJ, Pal S, Siddique D, Hyare H, Macfarlane R, Joiner S et al. Clinical presentation and pre-mortem diagnosis of variant Creutzfeldt-Jakob disease associated with blood transfusion: a case report. Lancet 2006; 368: 2061–2067. 17161728 10.1016/S0140-6736(06)69835-8

[bib124] Bellingham SA, Coleman BM, Hill AF. Small RNA deep sequencing reveals a distinct miRNA signature released in exosomes from prion-infected neuronal cells. Nucleic Acids Res 2012; 40: 10937–10949. 22965126 10.1093/nar/gks832 PMC3505968

[bib125] Arellano-Anaya ZE, Huor A, Leblanc P, Lehmann S, Provansal M, Raposo G et al. Prion strains are differentially released through the exosomal pathway. Cell Mol Life Sci 2015; 72: 1185–1196. 25227242 10.1007/s00018-014-1735-8 PMC11113346

[bib126] Polanco JC, Scicluna BJ, Hill AF, Gotz J. Extracellular vesicles isolated from the brains of rTg4510 mice seed Tau protein aggregation in a threshold-dependent manner. J Biol Chem 2016; 291: 12445–12466. 27030011 10.1074/jbc.M115.709485 PMC4933440

[bib127] Zhuang X, Xiang X, Grizzle W, Sun D, Zhang S, Axtell RC et al. Treatment of brain inflammatory diseases by delivering exosome encapsulated anti-inflammatory drugs from the nasal region to the brain. Mol Ther 2011; 19: 1769–1779. 21915101 10.1038/mt.2011.164 PMC3188748

[bib128] El Andaloussi S, Lakhal S, Mager I, Wood MJ. Exosomes for targeted siRNA delivery across biological barriers. Adv Drug Deliv Rev 2013; 65: 391–397. 22921840 10.1016/j.addr.2012.08.008

[bib129] Alvarez-Erviti L, Seow Y, Yin H, Betts C, Lakhal S, Wood MJ. Delivery of siRNA to the mouse brain by systemic injection of targeted exosomes. Nat Biotechnol 2011; 29: 341–345. 21423189 10.1038/nbt.1807

[bib130] El-Andaloussi S, Lee Y, Lakhal-Littleton S, Li J, Seow Y, Gardiner C et al. Exosome-mediated delivery of siRNA *in vitro* and *in vivo*. Nat Protoc 2012; 7: 2112–2126. 23154783 10.1038/nprot.2012.131

[bib131] Khatua AK, Taylor HE, Hildreth JE, Popik W. Exosomes packaging APOBEC3G confer human immunodeficiency virus resistance to recipient cells. J Virol 2009; 83: 512–521. 18987139 10.1128/JVI.01658-08 PMC2612372

[bib132] Vlassov AV, Magdaleno S, Setterquist R, Conrad R. Exosomes: current knowledge of their composition, biological functions, and diagnostic and therapeutic potentials. Biochim Biophys Acta 2012; 1820: 940–948. 22503788 10.1016/j.bbagen.2012.03.017

[bib133] Naslund TI, Paquin-Proulx D, Paredes PT, Vallhov H, Sandberg JK, Gabrielsson S. Exosomes from breast milk inhibit HIV-1 infection of dendritic cells and subsequent viral transfer to CD4+ T cells. AIDS 2014; 28: 171–180. 24413309 10.1097/QAD.0000000000000159

[bib134] Akao Y, Iio A, Itoh T, Noguchi S, Itoh Y, Ohtsuki Y et al. Microvesicle-mediated RNA molecule delivery system using monocytes/macrophages. Mol Ther 2011; 19: 395–399. 21102562 10.1038/mt.2010.254 PMC3034851

[bib135] van den Boorn JG, Schlee M, Coch C, Hartmann G. SiRNA delivery with exosome nanoparticles. Nat Biotechnol 2011; 29: 325–326. 21478846 10.1038/nbt.1830

[bib136] DeMarino C, Schwab A, Pleet M, Mathiesen A, Friedman J, El-Hage N et al. Biodegradable nanoparticles for delivery of therapeutics in CNS infection. J Neuroimmune Pharmacol 2016; (e-pub ahead of print). 10.1007/s11481-016-9692-7 PMC5205567 27372507

[bib137] Sampey GC, Meyering SS, Asad Zadeh M, Saifuddin M, Hakami RM, Kashanchi F. Exosomes and their role in CNS viral infections. J Neurovirol 2014; 20: 199–208. 24578033 10.1007/s13365-014-0238-6 PMC4378677

[bib138] Dias V, Junn E, Mouradian MM. The role of oxidative stress in Parkinson's disease. J Parkinsons Dis 2013; 3: 461–491. 24252804 10.3233/JPD-130230 PMC4135313

[bib139] Haney MJ, Klyachko NL, Zhao Y, Gupta R, Plotnikova EG, He Z et al. Exosomes as drug delivery vehicles for Parkinson's disease therapy. J Control Release 2015; 207: 18–30. 25836593 10.1016/j.jconrel.2015.03.033 PMC4430381

[bib140] Haney MJ, Zhao Y, Harrison EB, Mahajan V, Ahmed S, He Z et al. Specific transfection of inflamed brain by macrophages: a new therapeutic strategy for neurodegenerative diseases. PLoS One 2013; 8: e61852. 23620794 10.1371/journal.pone.0061852 PMC3631190

[bib141] Zhao Y, Haney MJ, Gupta R, Bohnsack JP, He Z, Kabanov AV et al. GDNF-transfected macrophages produce potent neuroprotective effects in Parkinson's disease mouse model. PloS One 2014; 9: e106867. 25229627 10.1371/journal.pone.0106867 PMC4167552

